# Validation of a Brazilian Portuguese Version of the Walking Estimated-Limitation Calculated by History (WELCH)

**DOI:** 10.5935/abc.20160004

**Published:** 2016-01

**Authors:** Gabriel Grizzo Cucato, Marilia de Almeida Correia, Breno Quintella Farah, Glauco Fernandes Saes, Aluísio Henrique de Andrade Lima, Raphael Mendes Ritti-Dias, Nelson Wolosker

**Affiliations:** 1 Hospital Israelita Albert Einstein, São Paulo, SP - Brazil; 2 Hospital das Clínicas da Faculdade de Medicina da Universidade de São Paulo, São Paulo, SP - Brazil; 3 Escola Superior de Educação Física - Universidade de Pernambuco, Recife, PE - Brazil; 4 Escola de Educação Física e Esporte da Universidade de São Paulo, São Paulo, SP - Brazil

**Keywords:** Questionnaires, Gait, Walking, Intermittent Claudication, Predictive Value of Tests, Disability Evaluation, Brazil

## Abstract

**Background:**

The Walking Estimated-Limitation Calculated by History (WELCH) questionnaire has been proposed to evaluate walking impairment in patients with intermittent claudication (IC), presenting satisfactory psychometric properties. However, a Brazilian Portuguese version of the questionnaire is unavailable, limiting its application in Brazilian patients.

**Objective:**

To analyze the psychometric properties of a translated Brazilian Portuguese version of the WELCH in Brazilian patients with IC.

**Methods:**

Eighty-four patients with IC participated in the study. After translation and back-translation, carried out by two independent translators, the concurrent validity of the WELCH was analyzed by correlating the questionnaire scores with the walking capacity assessed with the Gardner treadmill test. To determine the reliability of the WELCH, internal consistency and test-retest reliability with a seven-day interval between the two questionnaire applications were calculated.

**Results:**

There were significant correlations between the WELCH score and the claudication onset distance (r = 0.64, p = 0.01) and total walking distance (r = 0.61, p = 0.01). The internal consistency was 0.84 and the intraclass correlation coefficient between questionnaire evaluations was 0.84. There were no differences in WELCH scores between the two questionnaire applications.

**Conclusion:**

The Brazilian Portuguese version of the WELCH presents adequate validity and reliability indicators, which support its application to Brazilian patients with IC.

## Introduction

Peripheral artery disease (PAD) affects approximately 202 million people worldwide, contributing to the overall global morbidity and mortality.^1^ In Brazil, PAD affects approximately 10.5% of the population over the age of 18 years.^2^ Intermittent claudication (IC), the main symptom of PAD, affects approximately one-third of these patients.^3^ IC is described as cramp, pain, or tiredness affecting the lower limbs during walking which is relieved by a short period of rest.^4,5^ These symptoms impair the walking capacity, physical fitness^6^, and quality of life of these patients.^7,8^

Walking capacity has been used as an important clinical outcome in patients with IC^9,10^, and the walking tests, including the graded treadmill test, has been considered the gold standard for this assessment.^11,12^ However, because walking tests are more time consuming and require adequate facilities, they are not often used in the clinical setting. Therefore, the use of easy and fast methods such as questionnaires has been proposed to evaluate functional capacity in patients with IC.^11,13-15^

The Walking Estimated-Limitation Calculated by History (WELCH) questionnaire was recently proposed as a new simple and easily scored four-item questionnaire to assess walking impairment in patients with IC. Although the WELCH questionnaire has been validated in other languages^16,17^, it has not yet been translated and validated into the Portuguese language, limiting its use in Brazilian patients with IC. Thus, the aim of the present study was to analyze the psychometric properties of a Brazilian Portuguese version of the WELCH questionnaire in patients with IC.

## Methods

In all, 100 patients of both genders with IC symptoms were recruited by convenience sampling from the Vascular Unit of the *Hospital das Clínicas* of the University of São Paulo. Inclusion criteria were: (a) ≥ 50 years of age; (b) PAD stage II in one or both lower limbs according to Fontaine’s classification;^18^ c) ability to walk on a treadmill for at least two minutes at 3.2 km/h and 0% inclination; and d) limitations to the treadmill test due to IC symptoms. Patients with an ankle-brachial index (ABI) > 1.30 and/or non-compressible arteries on both sides were excluded. Eighty-four patients met all inclusion criteria and participated in the study.

The study was approved by the Institutional Review Board (process: 1973-14) and was performed according to international ethics standards conforming to the Declaration of Helsinki. All patients provided written informed consent to participate.

### Study design

After the translation and back-translation procedures, the Brazilian Portuguese version of the questionnaire was tested in all patients with IC. In addition, medical history, ABI,^19^ and walking capacity assessed with the Gardner-Skinner graded treadmill test^20^ were obtained. In order to analyze the test-retest reliability of the Brazilian Portuguese version of the WELCH questionnaire, a subsample of 17 patients was reevaluated after 7 days with the same procedures of the first evaluation.

### Translation of the WELCH questionnaire

The translation of the questionnaire was carried out by a qualified professional specialist in translations whose native language is Brazilian Portuguese, and who is fluent in English and experienced in the translation of manuscripts in the medical field. The translator was informed of the proposal of the study and the target population to whom the questionnaire would be applied. Additionally, the translator was advised to carry out a semantic translation and not just a literal one, as well as to use words that would cause the same impact in our cultural context, aiming at the reproduction of the same emotional response.

After translation of the questionnaire, the test phase was carried out evaluating the comprehension of the questionnaire by individuals with IC. In order to do that, an additional sample of 30 patients with IC (who did not participate in the process of determining the validity and reliability of the questionnaire) was selected. At this phase, individuals with IC were asked to comment on the questionnaire questions, pointing out difficulties and suggesting terms that would be easier to understand. Based on the patients’ comments, the questionnaire was then reanalyzed by a healthcare professional who made small alterations to improve its comprehension. Afterwards, the questionnaire was back-translated into English by a different bilingual translator who, similar to the first one, was also a qualified professional specialist in translations whose native language was Brazilian Portuguese, and who was also fluent in English and experienced in the translation of manuscripts in the medical field, both in the Portuguese and English languages. It is important to mention that the translator in charge of the back-translation was blinded to the original English version of the WELCH.

The authors then appraised the translated and back-translated versions through comparisons with the original text for correction of discrepancies and creation of a consensus version. In order to create this version, the semantic equivalencies (words with the same meaning) and idiomatic equivalencies (equivalent slang and colloquial expressions) were carefully preserved to present a simple and direct vocabulary.

### The WELCH questionnaire

The questionnaire answers were obtained through interviews and scored as previously described.^21^ In brief, each of the eight answers to the first three questionnaire items has a value ranging from 0 to 7, and each of the five answers proposed for the last item, which deals with usual walking speed, has a coefficient ranging from 1 to 5. The score is calculated as the sum of the values for the first three questionnaire items, minus one, multiplied by the coefficient of the final questionnaire item (walking speed). The WELCH score ranges from 0 to 100, with zero indicating a patient who can only walk for 30 seconds when walking slowly and who usually walks much slower than his or her relatives, friends, or people of the same age. A score of 100 would indicate a patient who can walk three hours or more, even when walking fast, and who usually walks faster than his or her relatives, friends, or people of the same age.

### Statistical analysis

All analyses were carried out with SPSS version 17 (IBM, Chicago, IL). The Gaussian distribution and the homogeneity of variance of the data were analyzed using the Shapiro-Wilk and Levene tests. Validity was determined by measuring the concurrent validity. Since a non-Gaussian distribution was observed, the relationship between the WELCH score and walking impairment (claudication onset distance and total walking distance) on the treadmill test was assessed using Spearman's rank correlation coefficient. Internal consistency, measured by Cronbach’s alpha, and test-retest reliability, analyzed with the intraclass coefficient of correlation and Bland-Altman limits of agreement, were calculated to determine the reliability of the WELCH. Values are presented as mean ± standard deviation for quantitative variables and as frequency for categorical variables. The level of significance for all inferential analyses was set at p < 0.05. 

## Results

The characteristics of the patients are shown in Table 1. They were mostly elderly (64.8 ± 8.8 years), male (77%), and hypertensive (86%).

**Table 1:** Characteristics of the patients

**Variable**	
Age (yrs)	64.8 ± 8.8
Men (%)	77.0
Weight (kg)	72.8 ± 10.5
Body mass index (kg/m2)	25.9 ± 3.3
Ankle-brachial index	0.61 ± 0.13
**Risk factors**	
Previous smoking (%)	69
Current smoking (%)	22
Hypertension (%)	86
Diabetes mellitus (%)	40
Dyslipidemia (%)	87
Obesity (%)	13
**Walking capacity**	
Claudication onset distance	156 ± 113
Total walking distance	397 ± 210
**Questionnaire**	
WELCH score	24 ± 21

Values are presented as mean ± standard deviation and frequency.

**Figure 1:**
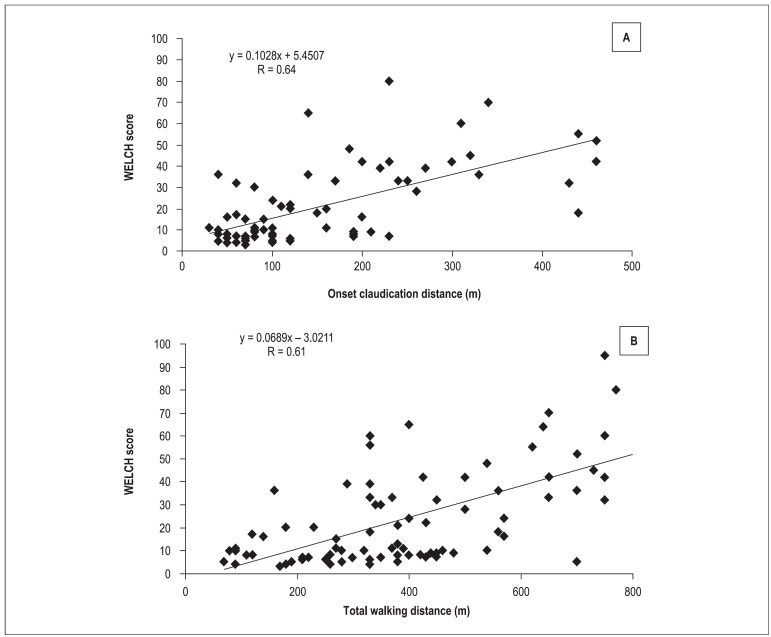
Scatterplots of the WELCH score versus claudication onset distance (A) and the WELCH score versus total walking distance (B).

The mean WELCH score was 24 ± 21. The mean claudication onset distance and total walking distance were 156 ± 113 m and 397 ± 210 m, respectively. There was a significant positive correlation between the WELCH score and the claudication onset distance (Figure 1A; r = 0.64, p = 0.01) and total walking distance (Figure 1B; r = 0.61, p = 0.01).

**Figure 2:**
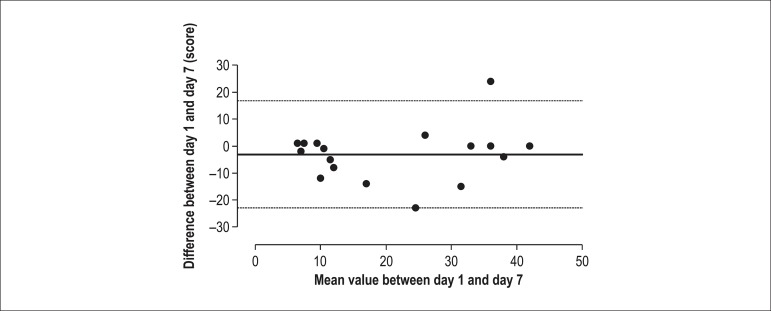
Bland and Altman plot of the total WELCH score (N = 17).

The internal consistency for the total WELCH score was 0.84, showing a sufficient homogeneity. The test-retest analysis indicated an intraclass correlation coefficient of 0.84 and a satisfactory agreement (bias = -3.11 ± 6.23 n.u; 95% limits of agreement: -22.92 to 16.69), with only 4.7% of the subjects out of the limits of agreement (Figure 2). 

## Discussion

The main finding of this study was that the Brazilian Portuguese version of the WELCH presented adequate validity and reliability indicators in a sample of Brazilian patients. This finding suggests that the Brazilian Portuguese version of the WELCH questionnaire could be useful to evaluate walking impairment in Brazilian patients with IC.

Although several questionnaires have been developed to assess walking impairment in patients with IC, the WELCH has been shown to be simpler and easy to score compared with others questionnaires.^21,22^ For concurrent validity (total WELCH score compared with treadmill testing) the Brazilian Portuguese WELCH score correlated significantly with the claudication onset distance (r = 0.64) and total walking distance (r = 0.61) obtained from a graded treadmill test. These coefficients are greater than those previously reported for the PAVK-86 questionnaire (r = -0.47),^23^ physical functioning subscale of the SF-36 questionnaire (r = 0.31)^24^ and three domains (distance, velocity, and stairs) of the Brazilian Portuguese version of the Walking Impairment Questionnaire (WIQ) (r = 0.30-0.43), validated in Brazilian patients with IC.^13^

The moderate positive correlation observed between the Brazilian Portuguese WELCH score and objectively measured walking capacity results was similar to the coefficients observed in the original French version (r = 0.61),^21^ which included a different constant-load treadmill protocol (constant load 3.2 km/h and 10% gradient for 15 minutes), and to the English version of the WELCH questionnaire (r = 0.59),^17^ which included the same treadmill protocol (Gardner-Skinner) used in this study. Thus, the WELCH score seems to present a moderate association with a variety of walking tests, providing information regarding different patterns of ambulation. However, it should be highlighted that in the English version of the WELCH, Tew et al^17^ found a strong positive correlation (r = 0.82) between the WELCH score and the six-minute walking test performance, indicating that the WELCH questionnaire may correlate better with tests that simulate physical activities during daily life.

The reliability of the WELCH in this study was determined with calculations of internal consistency and test-retest reliability. First, the internal consistency was 0.84 for the Brazilian Portuguese WELCH score, implying sufficient homogeneity of this questionnaire. Second, test-retest reliability analyzed by the intraclass coefficient correlation of the Brazilian Portuguese WELCH score was 0.84. In addition to the intraclass coefficient of correlation, we applied in our study the Bland-Altman limits of agreement method. In this analysis, we found satisfactory agreement (bias = -3.11 ± 6.23 n.u; 95% limit of agreement: -22.92 to 16.69), with only 4.7% of the subjects out of the limits of agreement. Taken together, these results indicate adequate reliability indicators in the Brazilian Portuguese WELCH in patients with IC. 

PAD affects approximately 10.5% of the Brazilian population aged more than 18 years,^2^ and assessment of walking capacity in these patients is useful to identify the presence of walking impairment. The practical application of the current study is that the Brazilian Portuguese WELCH version presents adequate validity and reliability indicators, and consequently, can be used in the clinical setting in Brazilian locations where the treadmill test cannot be performed. It is important to note that due to the low educational levels in patients who utilize the public health system, our version of the questionnaire uses an interview format, which is different than the self-completed format of other WELCH versions.^16,17,21,22^ Thus, in order to obtain similar results to those described in this study, the Brazilian Portuguese WELCH version should be applied using an interview format.

This study has some limitations. Its sample included patients with Fontaine Stage II PAD. Thus, the results cannot be extrapolated to patients at other stages of the disease (asymptomatic, stages III and IV). Our patients were already familiarized with the treadmill test since the test is routinely used in all patients with IC in our hospital. Thus, we cannot generalize our results to patients who do not have experience with objective measurements. Brazil is a country of continental dimensions and a diverse population. As such, the version of the WELCH that was translated in this study may not be valid for Brazilians from regions other than where the study was performed. Finally, the WELCH score was compared only with walking distances measured in a laboratory, whereas self-reporting tools appear to correlate better with community-based walking capacity tests, such as the six-minute walking test.^25^

## Conclusion

The Brazilian Portuguese version of the WELCH presents adequate validity and reliability indicators, which supports its application to Brazilian patients with IC.

## Appendix

Por favor, responda a cada um dos 4 itens seguintes, colocando um "X" no quadrado que melhor descreve a sua situação. Por favor, marque apenas um quadrado por item. Se você nunca executa a atividade, estime com seria se você a realizasse. Para os primeiros 3 itens, se você acha que não é capaz de realizar a tarefa especifica por pelo menos 30 segundos sem parar para descansar, por favor responda "impossível".

Para cada uma das três atividades seguintes, por quanto tempo você consegue, com facilidade, executar a tarefa em terreno plano e sem parar, quando você está...

**1/ ...andando devagar** (mais devagar que a velocidade usual de seus parentes, amigos, ou outras pessoas de sua idade)?

**Table:**

**Impossível**	**30 segundos**	**1 minuto**	**3 minutos**	**10 minutos**	**30 minutos**	**1 hora**	**3 horas ou mais**
				**X**			
0	1	2	3	4	5	6	7

**2/ ...andando normalmente** (velocidade igual à velocidade usual de seus parentes, amigos, ou outras pessoas de sua idade)?

**Table:**

**Impossível**	**30 segundos**	**1 minuto**	**3 minutos**	**10 minutos**	**30 minutos**	**1 hora**	**3 horas ou mais**
			**X**				
0	1	2	3	4	5	6	7

**3/ ...andando rapidamente** (mais rápido que a velocidade usual de seus parentes, amigos, ou outras pessoas de sua idade)?

**Table:**

**Impossível**	**30 segundos**	**1 minuto**	**3 minutos**	**10 minutos**	**30 minutos**	**1 hora**	**3 horas ou mais**
	**X**						
0	1	2	3	4	5	6	7

Em comparação com a velocidade de caminhada habitual de seus parentes, amigos ou pessoas de sua idade, você acha que você, pessoalmente, costuma andar... (assinalar apenas 1 opção)

**Table:**

Mutio mais devagar		1
Moderadamente mais devagar	**X**	2
Um pouquinho mais devagar		3
Na mesma velocidade		4

Muito Obrigado: Por favor, certifique-se de que assinalou uma opção em cada item.

Escore WELCH = [(4 + 3 + 1) - 1] x 2 = 14
